# Identification and Characterization of *Onchocerca volvulus* Heat Shock Protein 70 (*Ov*HSP70) as Novel Diagnostic Marker of Onchocerciasis in Human Urine

**DOI:** 10.3390/pathogens13040293

**Published:** 2024-03-30

**Authors:** Lum Abienwi Ambe, Elisabeth Limunga, Clarisse Engowei Mbah, Ngwewondo Adela, Ndumu Eric, Martha Ngoe, Bertrand Sone, Günter Lochnit, Julius Babila Tachu, Samuel Wanji, Anja Taubert, Carlos Hermosilla, Faustin Kamena

**Affiliations:** 1 Laboratory for Molecular Parasitology, Department of Microbiology and Parasitology, University of Buea, Buea P.O. Box 63, Cameroon; elisabethlimunga16@gmail.com (E.L.); ndumueric85@gmail.com (N.E.); marthangoe@yahoo.com (M.N.); mysonpalle1@gmail.com (B.S.); babila_tachu@yahoo.co.uk (J.B.T.); 2 Centre for Research on Health and Priority Pathologies, Institute of Medical Research and Medicinal Plants Studies (IMPM), Yaounde P.O. Box 13033, Cameroon; engoweiclarise@gmail.com (C.E.M.); adelafopezi@yahoo.fr (N.A.); 3 Protein Analytics, Institute of Biochemistry, Faculty of Medicine, Justus Liebig University Giessen, 35392 Giessen, Germany; guenter.lochnit@biochemie.med.uni-giessen.de; 4 Department of Microbiology and Parasitology, Faculty of Science, University of Buea, Buea P.O. Box 63, Cameroon; samwandji@gmail.com; 5 Research Foundation in Tropical Disease and Environment (REFOTDE), Buea P.O. Box 474, Cameroon; 6 Biomedical Research Center Seltersberg (BFS), Institute of Parasitology, Justus Liebig University Giessen, 35392 Giessen, Germany; anja.taubert@vetmed.uni-giessen.de (A.T.); carlos.r.hermosilla@vetmed.uni-giessen.de (C.H.)

**Keywords:** diagnosis, biomarker, filariasis, urine sample, antigen detection, neglected tropical disease

## Abstract

Despite several decades of mass drug administration and elimination-related activities, human onchocerciasis still represents a major parasitic threat in endemic regions. Among the challenges encountered by the elimination program is the lack of a suitable diagnostic tool that is accurate and non-invasive. Currently used methods are either invasive or not suitable for monitoring large numbers of patients. Herein, we describe the identification and characterization of *Onchocerca volvulus* heat shock protein 70 (*Ov*HSP70) as a novel diagnostic biomarker for human onchocerciasis, which can directly be detected in urine samples of infected patients. This nematode-specific antigen was identified through LC-MS after differential SDS-PAGE using urine-derived protein extracts from *O. volvulus*-infected patients in Cameroon. Polyclonal antibodies generated in rabbits after cloning and expression of *Ov*HSP70 in *Escherichia coli* reliably differentiated between urine samples from infected- and uninfected patients in a hypoendemic area of human onchocerciasis. These results provide an excellent basis for further development of a non-invasive and scalable diagnostic assay for human onchocerciasis using urine samples. Such a urine-based diagnostic assay will be of major importance for the elimination program of human onchcerciasis in endemic countries.

## 1. Introduction

Human onchocerciasis (river blindness) is amongst the five chemo-preventive Neglected Tropical Diseases (NTDs), and WHO’s goal under The Expanded Special Program for Elimination of Neglected Tropical Diseases (ESPEN) is to eliminate onchocerciasis with other NTDs by 2030 [1] It is the second cause of infectious blindness worldwide after trachoma [2], causing devastating skin diseases, which results in socioeconomic hardship and promotion of poverty in the affected communities living in endemic regions. 

Sub-Saharan Africa harbors about 99% of cases reported in 31 countries [3]. Currently, at least 217.5 million people live in areas endemic for human onchocerciasis [4] and about 244 million people in 30 countries require medical interventions in order to eliminate human onchocerciasis [5].

Disease control has been closely linked to regular treatments of patients, mapping and control of blackfly biomes. During the Onchocerciasis Control Program (OCP) era, mapping of the disease was mainly performed by entomological investigations [6]. Thereafter, the African Program for Onchocerciasis Control (APOC) era combined treatment and mapping, administering ivermectin in hyperendemic regions [7]. This approach succeeded in significantly reducing microfilaria (mf) loads in individuals to very low levels. At the end of their mandate, the ESPEN took over and changed the goal from control to elimination of human onchocerciasis and other NTDs by 2030 [5]. Within the frame of the new ESPEN strategy, antibody-based ELISAs and rapid diagnostic tests (RDTs) for the detection of *Ov*16 antibodies are mainly used as their mapping tool to diagnose for the disease in hypoendemic regions and areas that did not receive ivermectin treatments at all [8,9,10,11]. Unfortunately, antibody-based detection tools cannot differentiate between current or past infections since antibody levels generated against a pathogen/parasite remain high in individuals even after the disease is cleared [12]. Therefore, to carry out human onchocerciasis elimination mapping, accurate point-of-care diagnostics are needed to not only differentiate between infected and non-infected patients, but also assess the current infection status for an optimal anthelmintic treatment. Though much efforts have been put in place by control programs over past decades to eradicate this disease, a major impeding factor remains the lack of an appropriate diagnostic tool which can enable mapping and decision making to end mass drug administration (MDA) and therefore to minimize the development of resistance [5].

Presently, control is achieved mainly by the repetitive distribution of microfilaricidal ivermectin, which is the only approved drug against human onchocerciasis. In total absence of a macrofilaricide, the parasite continuously reproduces in humans even after the administration of ivermectin. Strikingly, in cases of co-endemicity with loiasis, caused by the closely related filarial nematode *Loa loa*, severe adverse events (SAEs) such as encephalopathies, which may lead to death, have been reported [13,14]. In addition, increasing reports on the development of resistance to ivermectin renders control highly problematic in hyperendemic geographic areas [15].

An *O. volvulus* infection is commonly diagnosed by detecting the presence of microfilariae in skin snips by light microscopy, which is the gold standard diagnostic test. As filarial diseases may often occur without detectable microfiladermia, this makes parasitological confirmation of infection extremely unreliable. As efforts are being made towards the elimination of this disease, one critical existing need is the development of improved diagnostics which can facilitate mapping and decision making [4]. This is because affordable skin snip microscopy shows low sensitivity, especially when microfilaria densities are as low as seen in hypoendemic areas with consistent mass drug administration. Moreover, skin snip is highly invasive, painful and has been rejected by entire communities, so it is not appropriate for use in elimination programs [16,17]. Thus, improved non-invasive diagnostic tools are needed for human onchocerciasis elimination programs in order to identify hypoendemic areas that were excluded from prior control programs and to determine when transmission has been interrupted. The absence of a widely accepted early, sensitive and objective laboratory diagnosis by control programs to support disease evaluation, monitor disease progression, direct treatment and identify affected individuals is a major problem hindering the activities of control programs worldwide. A sensitive method that can identify the presence of adult nematodes in patients will be useful in diagnosing those patients with low mf loads that are missed by classical microscopic methods, thereby significantly improving diagnosis. 

Testing the population with a diagnostic tool that detects the presence of adult *O. volvulus* before anthelmintic treatment is a necessity since clearing mf by routine treatments does not stop disease progress. Importantly, even after anthelmintic treatment, improved diagnostics are still needed to monitor the effectiveness of treatments in patients. 

Polymerase chain reaction (PCR) is the most sensitive and accurate diagnostic method used in onchocerciasis diagnosis. However, due to high cost and the need for special expertise and instrumentation, it is not suitable for screening large populations [18]. Although serological assays, such as the *Ov*16 ELISA for antibody detection, represent a cost-effective and easily deployable diagnostic approach [19], it shows lower performance in hypoendemic areas than in hyper- and meso-endemic areas [20]. For this reason, this serological method may not be capable of accurately assessing the infection status in all endemic areas.

Adult *O. volvulus* nematodes are routinely detected by the presence of palpable nodules in patients, especially in bony areas [21,22]. However, frequently, additional nodules are located deeper in tissues and therefore may be missed [23]. Moreover, a substantial underestimation of nodules is assumed to occur in people with more subcutaneous fat and/or who may have personal privacy reticence on a full medical examination. By detecting a parasite-specific protein rather than antibodies, a current infection can unequivocally be diagnosed [19,24]. Moreover, antigen detection assays generally require antibodies, which can be selected for precise specificity and produced in unlimited quantities for their use in diagnostic assays.

Urine as a waste material represents an ideal biological fluid for routine non-invasive diagnosis. It is easily collected and directly reflects the global state of an individual. Also, it allows monitoring of biological responses to drug therapy [25], can be used for measuring health and well-being, and represents an important tool for clinical diagnosis. Urine samples can also be donated with ease for diagnosis by patients since it is seen as valueless compared to other body fluids like blood, semen or vaginal secretion, which are considered as very precious fluids. 

Biomarkers to assess the infection status of treated individuals with anti-filaricidal activity are needed but currently lacking. Some efforts have been employed to search for biomarkers in urine that can distinguish between *O*. *volvulus*-infected and non-infected individuals [21,22,23,24]. More specifically, antigen biomarker mining from patient urine has been carried out using samples from donors with palpable nodules and mf densities over 50 mf/mg in samples collected over 20 years [25].

Heat shock protein 70 kDa (HSP70) belongs to a highly evolutionarily conserved protein family which has been described to include some of the most potent immunogenic molecules in a diverse number of parasitic organisms [26]. HSP70 has been shown to be constitutionally expressed by various filarial nematode species in both larval and adult stages including *Brugia pahangi* [27], *Brugia malayi* and *O. volvulus* [28,29]. It has been described as a vaccine candidate for human onchocerciasis based on a 12 amino acid region identified at the C-terminus of the protein rendering this segment unique to *O. volvulus* [30].

In this study, *O. volvulus*-specific HSP70 (*Ov*HSP70) was detected in urine samples of infected patients (mf+ by light microscopy) following proteomics analysis. The protein was cloned, expressed, purified and then used to immunize rabbits for specific antibody production. *Ov*HSP70-specific polyclonal antibodies were successfully used to differentiate between *O. volvulus*-infected and non-infected people in a field study in Cameroon. The current results demonstrate the applicability of *Ov*HSP70 as a novel biomarker for the diagnosis of human onchocerciasis and provide an important basis for further investigations to confirm this approach under field conditions.

## 2. Materials and Methods

### 2.1. Extraction and Identification of OvHSP70 from Patient Urine

#### 2.1.1. Urine Collection and Protein Precipitation

Urine collection from *O. volvulus*-infected patients as well as uninfected controls was conducted in September 2020 to identify antigenic biomarkers of *O. volvulus* in patient urine samples. After a skin snip-based light microscopy test [31] to determine the presence of *O. volvulus* microfilariae, each consenting participant, males and females at least 5 years old who have been residing in the area for at least 5 years, was requested to provide 10 mL of midstream urine in sterile leak-proof screw-capped vials. Urine was collected from study participants (*n* = 37) residing in four (4) villages in the Bafia Health District, a human onchocerciasis endemic area located about 100 km away from Yaounde and adequately transported at 4 °C to the laboratory. Urine samples were also collected from several controls, i.e., individuals residing in Yaounde and Buea (non-endemic areas for human onchocerciasis), (*n* = 37) and also from Europeans from Germany who have never been to Africa. 

#### 2.1.2. Acetone Precipitation of Urine Proteins

Proteins in urine samples were precipitated following a protocol described previously [32]. Briefly, a pool (100 µL of each sample) of urine from positive onchocerciasis patients (37 samples) and another from controls (*n* = 37) was made and inherent proteins were precipitated with acetone at a ratio of 1:5 [sample: cold acetone, (*v*/*v*)]. This mixture was incubated at −20 °C overnight followed by centrifugation for 10 min at 20,000× *g*. The protein pellets were solubilized in 8 M urea by sonication, and protein concentration was determined by Bradford assay [33].

#### 2.1.3. SDS PAGE

For analytical purposes, 3 mg of proteins was separated per slot via 12% standard sodium dodecyl sulfate polyacrylamide gel electrophoresis (SDS-PAGE) [34] alongside SIGMA low-molecular-weight markers. After staining with Coomassie blue, bands uniquely present in infected cases but absent in uninfected controls were identified with the help of the pre-stained protein standard.

For preparative purposes, proteins were run in a 12% mini-gel and subsequently stained by the colloidal blue method [35]. Unique bands present in the lane loaded with urine proteins from infected cases were excised using a sharp scalpel for in-gel digestion. The excised gel fragments were washed for 15 min in distilled water and the process was repeated 10 times. The gel fragments were cut into small pieces and then analyzed by mass spectrometry (MS). 

#### 2.1.4. In-Gel Protein Digestion

Proteins were subjected to reducing conditions by incubating the gel bands in 10 mM DTT/100 mM Ambic (ammonium bicarbonate) solution at 56 °C for 1 h. Then, the proteins were carbamidomethylated by treatments with 55 mM iodoacetamide/100 mM Ambic for 1 h at room temperature (RT) in the dark. Enzymatic digestions were performed by adding sequencing-grade porcine trypsin (1:50, Promega, Madison, WI, USA, 37 °C overnight). Tryptic peptides were extracted three times with 200 mL of ACN/water (1:1) solution. Extracts were dried via speed vacuum, resuspended in 50 mL of 0.1% formic acid and then stored at −20 °C until use for analysis by LC–MS/MS.

#### 2.1.5. LC/MS

The peptide samples obtained from proteolytic digestion were analyzed using an Agilent 1100 capillary LC (Palo Alto, CA, USA) interfaced directly to a LTQ linear ion trap mass spectrometer (Thermo Electron, San Jose, CA, USA). Mobile phases A and B were H_2_O/0.1% formic acid and ACN/0.1% formic acid, respectively. The peptide samples were loaded for 50 min using positive N2 pressure on a PicoFrit 8 cm by 50 mm column (New Objective, Woburn, MA, USA) packed with 5 mm diameter C18 beads. Peptides were eluted from the column into the mass spectrometer during a 60 min linear gradient from 5% to 60% of total solution composed of mobile phase B at a flow rate of 200 mL per min. The instrument was set to acquire MS/MS spectra on the 9 most abundant precursor ions from each MS scan with a repeat count of 1 and a repeat duration of 5 s. Dynamic exclusion was enabled for 200 s. Raw tandem mass spectra were converted into the mzXML^®^ format and then into peak lists using the ReAdW^®^ software version 4.3.1 followed by the mzMXL2Other^®^ software. The peak lists were then searched using Mascot 2.5 (Matrix Science, Boston, MA, USA) which described each protein read by its Protein FDR Confidence, Accession number, protein description, Exp. q-value, percentage coverage, number of peptides, unique peptides, PSMs, number of amino acids, molecular weight (kDa), calculated PI, mascot score and protein groups. 

#### 2.1.6. Bioinformatics Analysis

Using the data obtained from the MS analysis, the protein ID list provided by UniProt was used to search for the protein sequences on the UniProt^®^ database. Sequence similarities corresponding to filarial *O. volvulus* molecules were considered candidates of parasite-specific proteins if they showed greater than 90% similarity to *O. volvulus*. Based on the above, *Ov*HSP70 appeared as the top hit from the list of candidates. The UniProt amino acid sequence of the full-length *Ov*HSP70 protein was then introduced onto Protparam^®^ (http://web.expasy.org/protparam/ accessed on 29 July 2021) [36], an online database to obtain detailed characteristics of the selected protein [37]. 

### 2.2. Cloning, Expression and Purification of Recombinant OvHSP70 Antigen

The protein sequence was reverse-translated on the EMBOSS server on EMBL (https://www.ebi.ac.uk/Tools/st/emboss_bactranseq/ accessed on 4 August 2021) [38] to obtain the DNA sequence. The DNA sequence was then optimized for expression in *E. coli* k13 strain [39]. The gene sequence coding for the *Ov*HSP70 was outsourced to Synbio USA (Synbio Technologie LLC, NJ, USA) for synthesis and assembled into pET-15b (Synbio Technologie LLC, NJ, USA) vector in-frame to an N-terminal 6xHis tag with *XhoI* and *Nde I* flanking restriction enzyme cutter regions. The construct (Supplementary Figure S1) was used to transform *E*. *coli* BL21 DE3 PlysS cells. 

Protein expression was induced with 1 mM IPTG at 37 °C for 4 h, shaking at 225 rpm and protein expressed as a 6xHis fusion protein. The cells were then harvested by centrifugation at 4000× *g* for 30 min at 4 °C. 

The expressed protein was purified by affinity chromatography, using Ni-NTA agarose beads (Sigma-Aldrich, Darmstadt, Germany). Briefly, the harvested cells were washed in sodium chloride Tris EDTA (STE) buffer and lysozyme was added to a final concentration of 1 mg/mL. The suspension was then incubated on ice for 30 min while vortexing at intervals to promote cell lysis. Then, an ice-cold Triton buffer was added, and the cells were incubated on ice for another 30 min while vortexing. The cells were removed by centrifugation at 17,000× *g* for 10 min at 4 °C, 2 mL of a pre-equilibrated agarose Ni-NTA slurry was added to the supernatant and agitated for 1 h at 4 °C. The Ni-NTA-agarose/protein complex was sedimented by centrifugation (Eppendof 5810R) for 2 min at 510× *g* at 4 °C and washed 5× with a binding buffer. The *Ov*HSP70 protein was then eluted 4 times with an elution buffer. The different fractions were analyzed using SDS PAGE and Western blotting with addition of anti-His tag antibodies (Sigma-Aldrich, Darmstadt, Germany). Fractions containing the protein of interest were pooled and analyzed via SDS PAGE.

### 2.3. Rabbit Immunization and Production of Polyclonal Anti-OvHSP70 Antibodies

To generate polyclonal antibodies against the recombinant *Ov*HSP70 protein, a New Zealand rabbit was purchased (*n* = 1) at infancy from local vendors, acclimatized in a clean and ventilated cage with an isolated stable facility while supplying them with food and water ad libitum. During the period of acclimatization, the animal received anthelmintic treatments and was treated with antibiotics to clear off any eventual or suspected parasitic or bacterial infection. The rabbit was primed with 150 mg of the recombinant antigen homogenized in 300 µL of TitreMax Adjuvant^®^ (Sigma-Aldrich, Germany) by injecting the solution intramuscularly. The animal was then boosted twice with 75 mg of antigen in TitreMax Adjuvant^®^ 2 weeks after the first immunization, and subsequently 2 weeks after the second immunization. The animal was closely monitored for any undesirable reactions until the end of the stabling period. Every 14 days, 2 mL of blood was collected from the immunized animal to monitor antibody generation via Western blotting (WB) and indirect ELISA assays. At each time point of blood collection, serum was prepared by centrifuging blood samples for 15 min at 2500 rpm. The serum was collected in a separate tube, aliquoted in 1:2 glycerol and stored at −20 °C until further use. At day 100 post immunization, the animal was sacrificed, blood collected from the aorta and processed accordingly to obtain the final serum. 

### 2.4. Western Blotting (WB)

The reactivity of the polyclonal antibodies was evaluated using recombinant *Ov*HSP70 protein in Western blotting analyses. The electrophoresed protein was electroblotted onto nitrocellulose membranes as described before [40]. After blocking excess binding sites on the membrane using a blocking buffer (5% BSA in PBS, 0.1% Tween-20), antigen samples were incubated in a solution of the anti-*Ov*HSP70 polyclonal sera (1:2000) at 4 °C overnight. Then, secondary mouse anti-rabbit IgG-HRP antibodies (SIGMA, Germany) were employed at 1:3000 for 2 h at RT. All antibody dilutions were performed in washing buffer (PBS, 0.1% Tween-20). Reactive antigen bands were finally revealed in the dark using diaminobenzidine as enzyme substrate.

To assess for cross-reactivity of the polyclonal antibody, total cell lysate was obtained from the worm by macerating 2 mg of each helminth on ice in a lysis buffer for 2 min, after which the Laemmli buffer was added to the samples and boiled for 10 min at 95 °C. Samples were then kept in the freezer until used in Western blot as described above.

### 2.5. Indirect OvHsp70 ELISA

To evaluate the kinetics of rabbit antibody production against the *Ov*Hsp70 protein, each serum sample was tested by indirect ELISA. A volume of 50 µL of the purified *Ov*Hsp70 protein (48.85 ng) was used to coat the well of an ELISA plate using a coating buffer (overnight at 4 °C). The plate was washed 3 times in PBS-T (Sigma-Aldrich, Germany) the following day, then incubated in 5% bovine serum albumin (BSA, Sigma-Aldrich, Germany) for 2 h at RT. Thereafter, 100 µL of antisera was supplemented to the coated wells and incubated overnight at 4 °C while shaking at 200 rpm. The plate was then washed 4 times with PBS-T and incubated with 200 µL of a mouse anti-rabbit HRP (1:5000) (Sigma-Aldrich, Germany) secondary antibody for 2 h at RT. At the end of incubation, the plate was washed 6 times in PBS-T and reactions were visualized with TMB for 5 min. The reaction was stopped with 200 µL of 3 M HCl and then quantified by an ELISA plate reader at a wavelength of 450 nm. A graph of optical density (OD) against immunization time was plotted using Qtiplot 0.9.8.3-3^®^.

### 2.6. Filter Retardation Assays (FRAs)

FRAs were performed to determine the diagnostic performance of the generated antibodies. Each urine sample was spotted in triplicates (500 µL on each spot) on a nitrocellulose membrane with 0.2 µm pores (OE66, Schleicher and Schuell, Germany) using the slot blot filtration apparatus (Bio Rad, Germany). Membranes were blocked in Phosphate-buffered saline containing 5% BSA. Proteins retained on the filter membrane were incubated in anti-*Ov*HSP70 polyclonal antibodies (1:2000, overnight) and then in anti-rabbit secondary antibodies (Sigma-Aldrich, Germany) conjugated to horseradish peroxidase at 1:5000 to reveal the antigen/antibody reaction. To determine the diagnostic performance of the anti-*Ov*HSP70 antibodies, 136 mf-positive samples (119 palpable nodule negative), 30 nodule-positive but mf-negative samples, 30 mf-negative and 6 European samples were used for the analysis.

The signal intensities of the spots were quantified by densitometric analysis using the Fiji imaging software ImageJ^®^ (https://fiji.sc/, accessed on 23 January 2024) according to the non-NIH method [41] to obtain numerical values corresponding to the signal intensity per spot. The signal intensities were normalized with respect to the recombinant protein as standard positive control and the resultant values were obtained as ratios of the standard. The background was subtracted from each value in order to differentiate between negative and positive signals. These values were then used to calculate the diagnostic performance of the antibody, relative to the skin snip light microscopy analysis.

### 2.7. Statistical Analyses

The normality of distributions was assessed using the Shapiro–Wilk test. For non-Gaussian distributions, data were expressed as median with interquartile ranges and were compared using non-parametric tests. Comparisons of more than two groups were performed using the Kruskal–Wallis test (with Dunn’s or Tukey’s correction for multiple comparisons) for independent groups as appropriate. Diagnostic sensitivity and specificity as well as other diagnostic accuracy parameters were calculated as previously described [42,43]. Scatter plots were generated using R version 4.3.1^®^.

## 3. Results

### 3.1. Identification of OvHSP70 in Urine of Infected Patients

**Figure 1:**
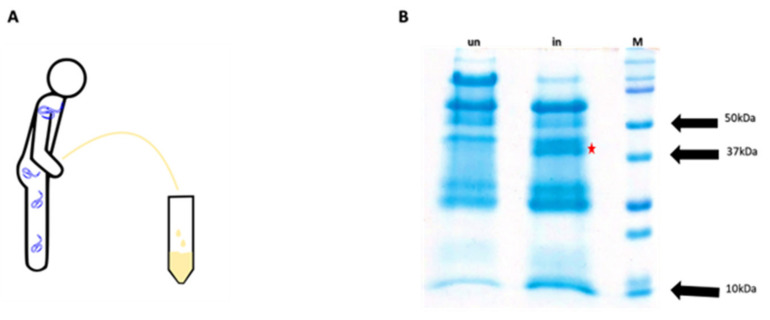
Sample collection and SDS gel of urine samples: Urine samples were collected (**A**) and proteins separated via SDS PAGE to identify unique bands in samples of *O. volvulus*-infected patients (**B**). The red asterisk in (**B**) indicates a unique protein band of approximately 37 kDA mass in a representative SDS PAGE gel in a pool sample of 37 human onchocerciasis-derived urines (in = infected) which is absent in a pool of 37 healthy (un = uninfected) urine donors from Yaounde and Buea; M: protein ladder.

Urine samples of *O. volvulus*-infected patients were pooled and submitted to protein precipitation by acetone treatments. Pooled urine samples from uninfected patients from non-endemic regions were used as negative controls. Precipitated proteins were separated on SDS-PAGE. A protein band with a mass of approximately 37 kDa was exclusively present in samples of infected patients but not in uninfected controls (Figure 1B). It was excised and analyzed using LC-MS. Sequencing results generated several protein candidates; two of them, which referred to the *Ov*HSP70, showed good mascot scores and high peptide coverage (Table 1). A previous report by Taylor and coworkers [30] suggested that despite its high homology to host HSP70, *Ov*HSP70 contains a short region of high species specificity. Based on this amino acid stretch of high species specificity, *Ov*HSP70 has been previously used as a vaccine candidate [30]. These results together with previous observations prompted us to focus on *Ov*HSP70 as our main candidate for further investigation.

### 3.2. Expression and Purification of OvHSP70; Generation of Polyclonal Anti-OvHSP70 Antibodies

**Figure 2:**
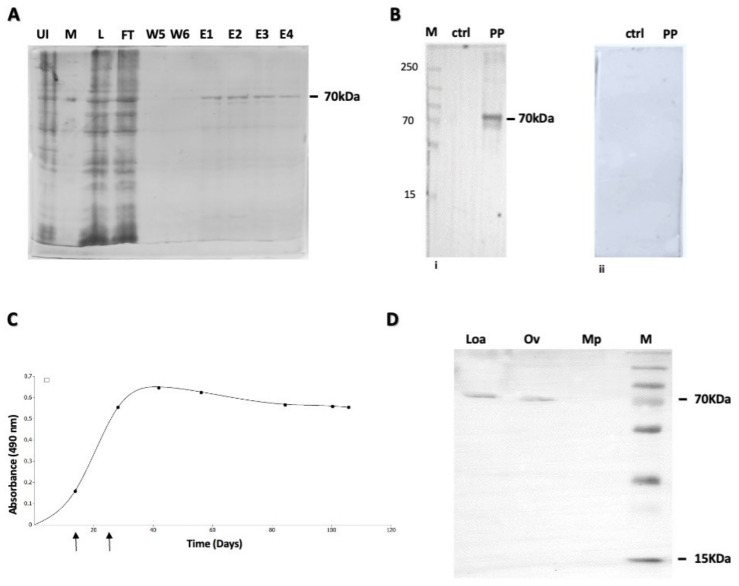
Recombinant expression of *Ov*HSP70, polyclonal antibody generation and cross-reactivity testing: (**A**) Purification of recombinant *Ov*HSP70 protein on Ni-NTA. E1–E4: elution fractions of purified antigen from the column, w5–w6: 5th and 6th washes, FT: flowthrough, L: cell lysate, M: protein ladder, UI: uninduced cells. (**B**) Polyclonal antibodies generated in rabbits against recombinant *Ov*HSP70 antigen specifically recognize the recombinant antigen at 70 kDa (i), whilst control protein (urine protein from Europeans) and sera (ii) prior to immunization show no reaction. (**C**) Kinetics of polyclonal antibody generation in the immunized rabbit. Indirect ELISA data indicate an exponential increase in antibody titer after boosting at days 14 and 28 indicated by arrows. (**D**) Assessing the cross-reactivity of the antibody on total cell lysates from *Onchocerca volvulus* (OV) and other helminths such as *Loa Loa* (Loa) and *Mansonella pestan* (Mp).

*Ov*HSP70 was successfully cloned, expressed and purified, thereby yielding a single protein band with a mass of approximately 70 kDa (Figure 2A). After purification, the protein was used to immunize a New Zealand rabbit. Monitoring of the generation of anti-*Ov*HSP70 antibodies in this rabbit by employing WB and ELISA using the purified recombinant antigen (Figure 2B,C) showed that antibody titers steadily increased upon boosting and reached a plateau at 42 days post immunization (Figure 2C). Furthermore, the cross-reactivity of the antibody was assessed by comparing the detection of *Ov*HSP70 in the presence of other helminths such as *Loa Loa* and *Mansonella pestans*. Total cell lysates generated from each of these parasites were ran through SDS-PAGE and detected using Western blot. The results show a clear cross-reactivity of the antibody with the antigen from *Loa Loa* but not with *Mansonella pestan* (Figure 2D).

### 3.3. Evaluation of Anti-OvHSP70 Specificity and Sensitivity Using Field Samples

**Figure 3:**
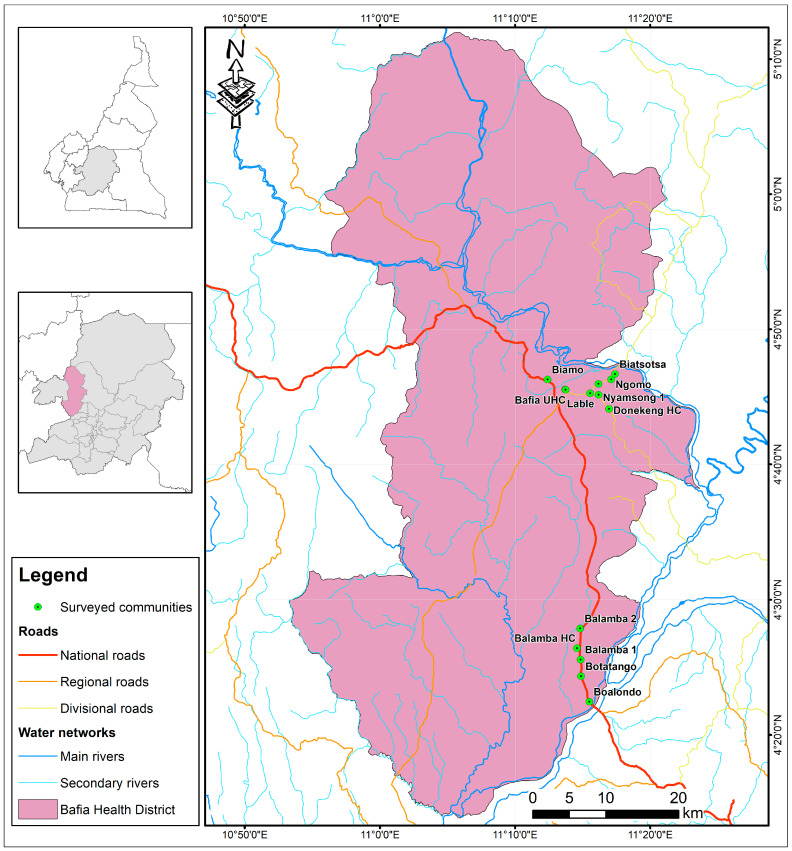
Geographic map of urine sample collection sites in the Bafia Health District, Centre Region of Cameroon, located along the river Sanaga.

**Figure 4:**
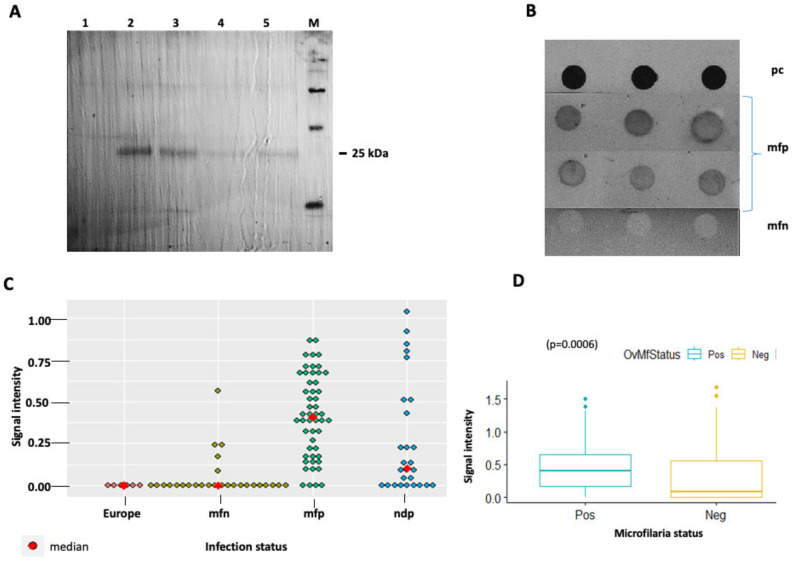
Western and dot blot assays evaluating the diagnostic potential of anti-*Ov*HSP70 polyclonal antibodies using field urine samples. (**A**) Individual patient samples (2–5) show a 25 kDa band on Western blots with the polyclonal anti-OvHSP70 antibody with no corresponding band on the control (1). (**B**) Alongside the recombinant antigen as a standard, samples and controls were tested in triplicates in dot blots. (**C**) Signal intensities were significantly different between mf-positive and -negative sample groups (*p* = 0.0006). (**D**) On single-sample level, some outliners were apparent in the mf-negative and nodule+/mf- group, unlike the European control showing no signals. pc: positive control, mfp: microfilaria-positive, mfn: microfilaria-negative, ndp: nodule-positive.

In order to evaluate the specificity and sensitivity of the generated polyclonal antibody, samples collected from infected patients were assayed first via Western blot and then via dot blot. Western blot positive samples showed a single band at 25 kDa. Following this result, we further assessed the antibody on a larger number of samples via dot blot. A total of 202 urine samples representing 30 mf negatives, 136 mf positives and 30 nodule-positives but mf negatives were collected from nine communities in the Bafia health district, a region endemic for human onchocerciasis (Figure 3). Of these samples, 196 represented people who were treated by Ivermectin. A total of six samples originating from European donors without any previous travel history to Africa were used as negative controls. All samples were subjected to filter retardation assays to concentrate protein therein on nitrocellulose membranes. The spotted samples were subsequently probed by anti-*Ov*HSP70 using dot blot assays. Quantification of the signal intensity was performed using non-NIH densitometry with the purified antigen serving as positive control. A representative blot of the experiment is shown in Figure 4B. In general, samples which tested positive for mf showed a significantly higher signal intensity than mf-negative samples (*p* = 0.0006). However, a few outliners of mf negatives and nodule positive but mf negative samples also revealed high signal intensities (Figure 4C).

Overall, the sensitivity of the anti-*Ov*HSP70 antibodies with respect to light microscopy was 87%. Pairwise comparison between different groups revealed the following specificities: 70% between mf positive and mf negative samples and 100% between mf positive and the European control group (Table 2). The specificity and sensitivity of *Ov*HSP70 detection in urine samples using the newly generated polyclonal antibody were 70% and 87%, respectively.

### 3.4. Increased Number of Ivermectin Treatments Reduces the Intensity of OvHSP70 Signals in Urine of Patients

**Figure 5:**
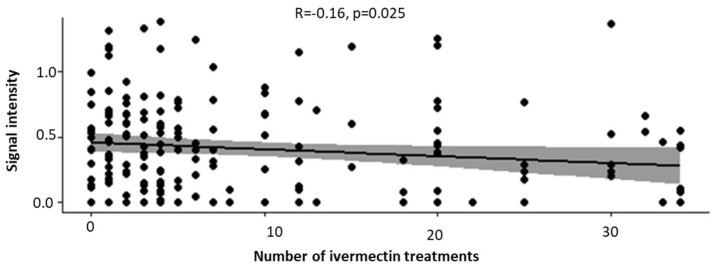
Correlation between signal intensity and number of ivermectin treatments: repeated ivermectin medication reduced the signal intensity for *Ov*HSP70 in dot blots. Shaded area represents the 95% confidence interval around the line of best fit.

Since the samples were collected from an endemic area that was part of the community-based mass drug administration (which includes administration of ivermectin at least once annually) for over 15 years, we decided to investigate whether the intensity of *Ov*HSP70 signals was affected by the regular treatment with this anthelmintic drug. We found a trend of negative correlation between *Ov*HSP70 signal intensity and the number of ivermectin treatments in participants of the community (*p* = 0.025, Figure 5). This may be an indication that *Ov*HSP70 detection could potentially serve as a marker of ivermectin treatment efficacy.

## 4. Discussion

Human onchocerciasis remains a major problem in endemic areas, and despite the implementation of mass drug administration (MDA) programs that started many decades ago, the elimination goal is by far not achieved in Africa and also in some foci in Yemen [1]. One of the key issues in accurately monitoring the elimination progress is a reliable diagnostic method with adequate sensitivity, specificity and relative ease of collection of sample types with good acceptance by the local community. Several diagnostic methods are currently available, such as light microscopy after skin snip [15,31] and an ELISA test to detect *Ov*16 antibodies in children below 10 years of age [44,45,46].

Taking epidermis biopsies for microscopy analysis is painful for patients and carries some risks of transmitting blood-borne infections, factors which caused a low tolerance or even general rejection by entire communities in endemic areas [15,47]. Of note, despite the high specificity of skin snip assays, this method is not reliably applicable for the assessment of transmission interruption since Mectizan™ treatments rapidly reduce the number of microfilariae present in the skin to very low levels [48]. Therefore, infections may be bypassed and misdiagnosed by this technique [49]. Communities exhibiting mf prevalence higher than 1% require treatment to interrupt transmission to blackflies, whilst below this threshold, the transmission route is considered as unsustainable.

Even though the detection of *Ov*16 antibodies is generally used as proof of exposure, it poses several challenges for control programs if they solely rely on these data. *Ov*16 IgG isotype detection greatly depends on IgG4 responses [45] which may take up to 14 months to be generated [50]. Considering this rather long period of time, *Ov*16 antibody detection may fail to diagnose a very recent exposure to *O. volvulus,* as intended by the eradication program. In addition, IgG-based antibody detection does not differentiate between past and present infections [44]. Moreover, approximately 20% of *O. volvulus*-infected individuals show some level of immunity, probably related to their genetic background, which restricts them from eliciting any humoral immune response to *Ov*16 antigens [45]. Furthermore, it has been shown that the *Ov*16-based ELISA has a rather low sensitivity to detect a skin snip-negative but infected individual (55% [46]).

Other tests to determine the presence of parasites intra vitam like the Ov-150bp-based PCR for humans [12,17], xenomonitoring [51], diethylcarbamazine patch tests [16] and nodule palpation are available but are inappropriate considering their drawbacks such as costs, low sensitivity, invasiveness or severe side effects [52].

In the current study, we aimed to develop an alternative diagnostic approach that is non-invasive, with a high patient acceptance and thus easy applicability for a large number of patients. We identified *Ov*HSP70 to be secreted in the urine of infected patients. Strikingly, polyclonal anti-*Ov*HSP70 antibodies raised in rabbits were able to detect a 25 kDa band on Western blots and reliably differentiate between urine samples from *O. volvulus*-infected and uninfected individuals on dot blots. These results indicate that *Ov*HSP70 represents an attractive and novel candidate for a diagnostic biomarker of onchocerciasis and for the development of a non-invasive diagnostic approach for the detection of human onchocercaisis directly from urine sample. The 25 kDa fragment is likely to be a degradation product of the full-length OvHSP70 as seen in the cell lysate from *O. volvulus* (Figure 2). Protein degradation could occur either in blood or as a result of handling the samples from the field or after a few rounds of freezing and thawing. Mining for diagnostic biomarkers for onchocerciasis has previously been achieved in patient serum [19,53,54] urine [55,56] and in silico [57,58].

Even though *Ov*HSP70 is not actively secreted via the classical secretory pathway, it is considered as an excretory secretory protein. Thus, it is actively expressed and released by the nematodes, most probably under stress [27]. The cross-reactivity of OvHSP70 to *Loa loa* cell lysate but not to *M. pestans* indicates that the polyclonal antibody may recognize some but not all helminths endemic in this region. However, in a further study, a more detailed analysis of the cross-reactivity will be necessary to better appreciate the specificity of the antibody. HSP70 of *O. volvulus* shares a high level of homology with human HSP70. However, previous studies have shown that despite an overall high homology, a parasite-derived HSP70 antigen includes a distinct short c-terminal region that clearly differs from human HSP70, especially in its non-conserved changes, secondary structures and B-cell epitopes [30]. This has conferred selective immune responses to non-homologous HSP regions differentiating human from parasitic HSP70 [27,28,29]. More importantly and irrespective of homology, *Ov*HSP70 has been proposed in the past as a biomarker for diagnostic purposes of human onchocerciasis albeit from blood samples [29]. To the best of our knowledge, this is the first time that *Ov*HSP70 was proven to be present in the urine of *O. volvulus*-infected patients. The current observation that polyclonal anti-*Ov*HSP70 antibodies were able to differentiate between infected and uninfected individuals in a field study using a dot blot experiment emphasizes the immense potential of this method to be further developed into a rapid and scalable test applicable for a larger population, e.g., for monitoring the elimination progress.

Although the current first methodical approach shows an encouraging specificity value of 70%, it does not meet the very high specificities observed for *Ov*16 ELISA (97% to >99.9%) [7,8,47] or the RDT (97% ± 98%) [10,46] both of which represent the currently recommended tests. Nonetheless, this result still represents a valuable proof of the principle that the *Ov*HSP70 antigen may be highly useful for the development of a non-invasive diagnostic assay that reliably detects the presence of the parasite in individuals, directly from the urine, rendering it more likely to be accepted by the residents than other methods.

Further analysis of our data showed a significant difference (*p* = 0.006) in the detection of *Ov*HSP70 depending on the parasitaemia status (mf+ or mf-). Additionally, there was a statistically significant negative correlation (*p* = 0.025, rho = −0.160876) between the number of Mectizan^®^ treatments and the detection of *Ov*HSP70 in the urine of patients, meaning that treatment-mediated reduction in parasite load can be controlled by this method. The current diagnosis of human onchocerciasis focuses the diagnostic need to verify suppression and blockage of transmission [59]. In cases of treatment, the sensitivity of the skin snip test also depends on the specific drug used and the time since treatment [46]. In the context of MDA evaluation, it is important to evaluate the impact of regular ivermectin treatments on test sensitivity, as proposed elsewhere [60]. Also, the presence of 119 mfp but palpable nodule-negative patients in this study might be an indication that some of the test-positive mfn are in fact ndp.

Strikingly, in the present study, *Ov*HSP70 was detected in the urine of patients from the Mbam valley, an area where the disease persists despite long-term treatments at a high prevalence and low mf intensities after more than 15 years of continuous ivermectin medication [61]. More interestingly, the urine samples used in this study were collected only four months after ivermectin administration.

Notably, currently available diagnostics fail to explicitly detect fertile female parasites in humans, which might continue reproduction once MDA is ended and which may therefore pose a risk for recrudescence. Moreover, not all adult nematodes need to be sterile or dead for transmission to be irreversibly interrupted. Ideally, we need to have a method at our disposal that detects all the different parasite stages, including adult female and male nematodes to actively interfere with mf release in vivo [25]. A sensitive, specific and operationally feasible biomarker assay for adult nematode infection is recommended [62] as there are currently no immunoassays commercially available to detect *O. volvulus* antigens in current infections for monitoring treatment efficacy and recrudescence of this disease. This will solve most of the challenges currently faced by the diagnostics of human onchocerciasis. The current study provides a solid basis for the development of such an assay using *Ov*HSP70 detection in urine samples. We believe that developing this assay will signify a major step forward in the monitoring and elimination of human onchocerciasis in Africa and elsewhere.

There is no doubt that to follow the aim of a functional *Ov*HSP70-based diagnostic assay, further research is needed. This will essentially include the generation of highly specific monoclonal anti-*Ov*HSP70 antibodies and evaluate the specificity and sensitivity of the tests in larger populations.

## Figures and Tables

**Table 1:** Characteristics of *OvHSP70* by mass spectrometry.

MS Description	Protein 1	Protein 2
**Checked**	FALSE	FALSE
**Protein FDR Confidence: Mascot**	High	High
**Master**	Master Protein	Master Protein
**Accession**	A0A3P6S5U3	A0A183HY13
**Description**	Uncharacterized protein OS = *Litomosoides sigmodontis* OX = 42,156 GN = NLS_LOCUS128 PE = 3 SV = 1	Uncharacterized protein OS = *Onchocerca flexuosa* OX = 387,005 GN = OFLC_LOCUS12375 PE = 3 SV = 1
**Exp. Q-value: Mascot**	0	0
**Coverage [%]**	11	12
**# Peptides**	6	8
**# PSMs**	6	8
**# Unique Peptides**	1	3
**# Aas**	680	1393
**MW [kDa]**	74.3	152.7
**calc. pI**	6.14	6.52
**Score Mascot: Mascot**	77	77
**# Peptides (by Search Engine): Mascot**	6	8
**# Protein Groups**	1	1

#: number of.

**Table 2:** Diagnostic potential of anti-*Ov*HSP70 antibodies.

Groups	Sensitivity (%)	Specificity (%)
mf+ versus mf-	87%	70%
mf+ versus European group	87%	a100

## Data Availability

All data have been provided in this manuscript.

## Supplementary Materials

The following supporting information can be downloaded at: https://www.mdpi.com/article/10.3390/pathogens13040293/s1, Figure S1: 6xHis-*Ov*HSP70 expression vector and nucleotide sequence of the full-length protein.
