# Supplementary material for: Identification and Characterization of Onchocerca volvulus Heat Shock Protein 70 (OvHSP70) as Novel Diagnostic Marker of Onchocerciasis in Human Urine
Source: Pathogens. 2024 Mar 30;13(4):293. doi: 10.3390/pathogens13040293 (PMC11053476; doi:10.3390/pathogens13040293)
Supplement: Supplementary file 1 [file pathogens-2868180-supplementary.pdf › pathogens-2868180-supplementary.pdf]

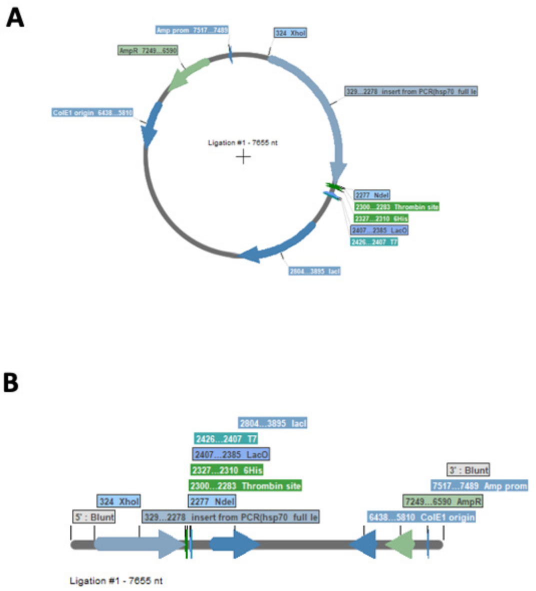

C

Sequence of the insert representing the full-length protein

Full-lenght *OvHSP70*
CATATGAGCAAAAACGCGATTGGCATTGATCTGGGCACCACCTATAGCTGCGTGGGC
GTGTTTATGCATGGCAAAGTGGAAATTATTGCGAACGATCAGGGCAACCGCACCACC
CCGAGCTATGTGGCGTTTACCGATACCGAACGCCTGATTGGCGATGCGGCGAAAAAC
CAGGTGGCGATGAACCCGCATAACACCGTGTTTGATGCGAAACGCCTGATTGGCCGC
AAATTTGATGATGGCAGCGTGCAGAGCGATATGAAACATTGGCCGTTTAAAGTGGTG
AACGCGGGCGGCGGCAAACCGAAAGTGCAGGTGGAATATAAAGGCGAAACCAAAACC
TTTACCCCGGAAGAAATTAGCAGCATGGTGCTGGTGAAAATGAAAGAAACCGCGGAA
GCGTTTCTGGGCCATGCGGTGAAAGATGCGGTGGTGACCGTGCCGGCGTATTTTAAC
GATAGCCAGCGCCAGGCGACCAAAGATAGCGGCGCGATTGCGGGCCTGAACGTGCTG
CGCATTATTAACGAACCGACCGCGGCGGCGATTGCGTATGGCCTGGATAAAAAAGGC
CATGGCGAACGCAACGTGCTGATTTTTGATCTGGGCGGCGGCACCTTTGATGTGAGC
ATTCTGACCATTGAAGATGGCATTTTTGAAGTGAAAAGCACCGCGGGCGATACCCAT
CTGGGCGGCGAAGATTTTGATAACCGCATGGTGAACCATTTTGTGGCGGAATTTAAA
CGCAAACATAAAAAAGATCTGGCGAGCAACCCGCGCGCGCTGCGCCGCCTGCGCACC
GCGTGCGAACGCGCGAAACGCACCCTGAGCAGCAGCAGCCAGGCGAGCATTGAAATT
GATAGCCTGTTTGAAGGCATTGATTTTTATACCAACATTACCCGCGCGCGCTTTGAA
GAACTGTGCGCGGATCTGTTTCGCAGCACCATGGATCCGGTGGAAAAAGCGCTGCGC
GATGCGAAAATGGATAAAGCGCAGGTGCATGATATTGTGCTGGTGGGCGGCAGCACC
CGCATTCCGAAAGTGCAGAAACTGCTGAGCGATTTTTTTAGCGGCAAAGAACTGAAC
AAAAGCATTAACCCGGATGAAGCGGTGGCGTATGGCGCGGCGGTGCAGGCGGCGATT
CTGAGCGGCGATAAAAGCGAAGCGGTGCAGGATCTGCTGCTGCTGGATGTGGCGCCG
CTGAGCCTGGGCATTGAAACCGCGGGCGGCGTGATGACCGCGCTGATTAAACGCAAC
ACCACCATTCCGACCAAAACCAGCCAGACCTTTACCACCTATAGCGATAACCAGCCG
GGCGTGCTGATTCAGGTGTATGAAGGCGAACGCGCGATGACCAAAGATAACAACCTG
CTGGGCAAATTTGAACTGAGCGGCATTCCGCCGGCGCCGCGCGGCGTGCCGCAGATT
GAAGTGACCTTTGATATTGATGCGAACGGCATTCTGAACGTGAGCGCGCAGGATAAA
AGCACCGGCAAACAGAACAAAATTACCATTACCAACGATAAAGGCCGCCTGAGCAAA
GATGAAATTGAACGCATGGTGCAGGAAGCGGAAAAATATAAAGCGGATGATGAAGCG
CAGAAAGATCGCATTGCGGCGAAAAACGCGCTGGAAAGCTATGCGTTTAACATGAAA
CAGACCATTGAAGATGAAAAACTGAAAGATAAAATTAGCGAAGATGATAAAAAAAAA
ATTCAGGAAAAATGCGATGAAACCGTGCGCTGGCTGGATGGCAACCAGACCGCGGAA
AAAGATGAATTTGAACATCGCCAGAAAGAACTGGAAGCGGTGTGCAACCCGATTATT
ACCAAACTGTATCAGAGCGCGGGCGGCATGCCGGGCGGCATGCCGGGCGGCATGCCG
GGCGGCATGCCGGGCGGCGCGCCGGGCGGCGGCAGCACCGGCGGCGGCCCGACCATT
GAAGAAGTGGAT**TAA**

Supplementary figure S1: Recombinant Protein construct for the OvHSP70 expression A. Circularized construct in pet15B, B. linearized form, C. Nucleotide sequence of OvHSP70
